# Doppler Ultrasound Diagnosis of Cystic Artery Pseudo-Aneurysm Causing Hemobilia

**DOI:** 10.5334/jbsr.3572

**Published:** 2024-05-03

**Authors:** Jose Taouk, Douglas Lacomblez, Pierre Bosschaert

**Affiliations:** 1 Radiology Intern, Clinique Saint-Pierre Ottignies, Belgium; 2 Clinique Saint-Pierre Ottignies, Belgium; 3 Clinique Saint-Pierre Ottignies, Belgium

**Keywords:** Cystic artery, Pseudo-aneurysm, Doppler ultrasound, Hemobilia, Cholangitis

## Abstract

*Teaching point:* Cystic artery pseudoaneurysm is a rare condition that should be considered in patients with unexplained abdominal pain, a history of gallbladder disorders, or signs of hemorrhage, and can be diagnosed by Doppler ultrasound.

## Case

A 76-year-old male patient presented with epigastric pain, vomiting and fever. He reported an episode of upper digestive bleeding with no proven explanation 10 years earlier.

**Figure 1:**
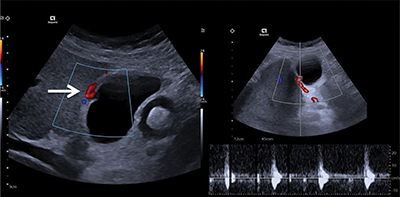
Doppler ultrasound showing a 5 mm anechoic micro-cavitary formation on the posterior wall of the gallbladder, presenting arterial flow, suggestive of a pseudo-aneurysm of the cystic artery.

Doppler ultrasound examination of the gallbladder showed a micro-cavitary formation of 5 mm on the posterior wall, presenting arterial flow, suggestive of a pseudo-aneurysm of the cystic artery. There were no signs of cholecystitis (Figure 1).

The patient showed rapid clinical deterioration with a drop in hemoglobin. Computed tomography (CT) was performed. The arterial phase showed an enhancement of the micro-cavitary formation observed sonographically, intracholecystic hematoma, and bile duct dilatation, thus confirming the suggested diagnosis.

**Figure 2:**
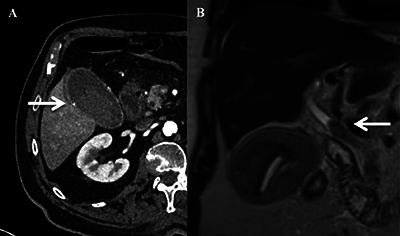
**(A)** Axial CT angiography confirming the diagnosis of cystic artery pseudoaneurysm (arrow). **(B)** Coronal T2-weighted MRI image showing an obstructing clot in the common bile duct (arrow).

Magnetic resonance imaging (MRI) clearly revealed the obstructive clot in the common bile duct (Figure 2A, B).

**Figure 3:**
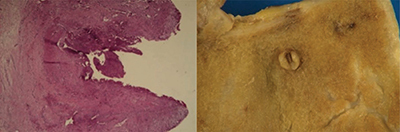
Histological examination and macroscopic appearance showing a pseudoaneurysmal rupture of the cystic artery protruding into the gallbladder lumen in a chronically inflammatory environment.

Histological examination after cholecystectomy confirmed a pseudoaneurysmal dilatation of the cystic artery protruding into the wall of the gallbladder in a chronically inflammatory environment (Figure 3).

## Comments

Cystic artery pseudoaneurysm (CAP) is a rare entity that typically occurs following cholecystitis and cholecystectomy. It may also be idiopathic.

Its pathogenesis remains unclear. For some authors, inflammation would cause damage to the vasa vasorum, weakening the elastic and muscular components of the arterial wall [1].

Rupture of the CAP generally manifests as hemobilia, which clinically results in a Quincke’s triad: upper gastrointestinal hemorrhage (45%), jaundice (60%), and epigastric pain (70%). However, in only 40% of cases do all three symptoms appear.

Rapid diagnosis by imaging is important because of the risk of complications such as hemorrhagic shock (digestive hemorrhage or significant hemoperitoneum) or acute pancreatitis.

Among the 77 cases reported in the literature, only three were initially diagnosed by Doppler ultrasound. These were macroaneurysms. Doppler ultrasound has the advantage of being easily available. Smaller lesions are difficult to detect as they can be hidden by a stone or located in an inflammatory thickening of the wall.

For larger entities, arterial flow recorded in the pseudoaneurysm sac may show the characteristic “Yin-Yang” sign, and spectral analysis a “To and Fro” flow pattern.

CT angiography is also a non-invasive imaging modality of choice for the diagnosis of CAP. The pseudoaneurysm may appear as a well-circumscribed, contrast-filled rounded pocket. Visualization of the neck is possible if the size of the lesion allows it.

An MRI can be useful in revealing a clot obstructing the common bile duct.

Traditionally, treatment by embolization or cholecystectomy is necessary. Arterial embolization followed by cholecystectomy is preferable for unstable patients.

It is important to emphasize the existence of this rare pathology occurring in a banal context and the use of Doppler ultrasound supplemented by CT angiography for rapid diagnosis and treatment in order to prevent associated potentially fatal complications.
